# Editors’ Book Table

**Published:** 1874-11

**Authors:** 


### Editors' Book Table.

[NOTE. — All works reviewed in the columns of the CHICAGO MEDICAL JOURNAL may be found in the extensive stock of W. B. KEEN, COOKE & CO., whose catalogue of Medical Books will be sent to any address upon request.]

*A Treatise on Food and Dietetics, Physiologically and Therapeutically Considered.* By F. W. PAVY, M.D., F.R.S., Fellow of the Royal College of Physicians, and Lecturer on Physiology at Guy's Hospital. Philadelphia: Henry C. Lea. 1874.

A summary of everything used for human food, from clay to turtle soup, by every nation in every clime. Dr. Pavy has ransacked the literature of gastronomy—not only ransacked but digested the most of it, and given us admirable instructions by means of which our own digestion may be made the more easy.

The author, moreover, has done much more than this, he has given us a history of food, its relations to the organism, its mode of appropriation, its sources of supply, its constituent elements, and an admirable classification of its principles and their chemical and physiological relations.

In addition to the consideration of the subject of food, in the restricted sense of edible matters, a considerable section of the work is devoted to that most essential of all its elements, water, together with those important adjuncts to the dietary of higher civilization, tea, coffee and cocoa, with the not less valuable wines, of all varieties and nationalities, and many analogous beverages.

The sections devoted to the description of food in its climatic and ethnological relations, present much that is interesting and suggestive, as are likewise the dietetic rules for infants and adults, and for hospitals.

The work will amply repay the reader, whether professional or general, and should find a place in the office of every physician, in the dispensary of every hospital, and would constitute a valuable addition to the household library. H.

*A Manual of Toxicology, including the consideration of the nature, properties, effects and means of detection of Poisons, more especially in their Medico-Legal Relations.* By JOHN J. REESE, M.D., Professor of Medical Jurisprudence and Toxicology in the University of Pennsylvania; Physician to St. Joseph's Hospital, etc., etc. Philadelphia: J. B. Lippincott & Co. 1874.

While the truth of the author's prefatory remark that "the literature of Toxicology is already voluminous," cannot be denied, his work cannot fail to be very acceptable to a large majority of his compatriots in the profession, for several reasons: first, in that it is a manual, and as such, devoid of much which the practical reader might regard as irrelevant; second, in that it is accessible to every one; and thirdly, that it is American.

Beginning with the best definition of "a poison," the author proceeds to draw a clear line of demarcation between matters essentially poisonous and those incidentally so; regarding which line public sentiment directed by the civil law is by no means well educated, and therefore will receive much enlightenment herein.

It is gratifying to perceive that our author is not a partisan of either the doctrine of absorption or of nervous sympathy as a means of inducing poisonous effects in the organism.

The chapter on compound poisons will be found to possess especial interest, in view of the practical application of which the antagonistic relations of different poisons are susceptible.

The advice to medical experts, and the delineation of the injustice to which they are so often subjected, should be thoroughly studied by every medical man who may be called upon at any time to appear upon the witness stand in this capacity. The impositions to which this class of witnesses is habitually subjected are narrated with the acumen derived from personal experience, and their condemnation must receive the endorsement of all who have similarly suffered.

Full details are given of the most approved methods of chemical analysis for the detection of poisons.

The chapters upon classification are full and explicit, and the exposition of the specific effects of various poisons as complete as the present state of the science permits. H.

*A Practical Treatise on the Diseases of Women.* By T. GAILLARD THOMAS, M.D., Professor of Obstetrics and Diseases of Women and Children in the College of Physicians and Surgeons, New York; etc., etc. Fourth edition, thoroughly revised. Philadelphia: Henry C. Lea. 1874.

Perhaps the highest praise which can be bestowed on any book is the demand on the part of the reading public for new editions—it certainly demonstrates one fact, that the book is adapted to their wants. This fact has been thoroughly established in the case of the book before us, of which three editions have been exhausted and a fourth issued within five years. Our transatlantic brethren have manifested their high appreciation of the work by translating it into German, as they will shortly do into French and Italian also.

We do not propose here to enter upon the superfluous task of making a detailed analysis of the contents of Dr. Thomas' excellent work. We say superfluous, because those already acquainted with the work could not thereby learn to estimate it more highly, and those to whom it is still unfamiliar can only do it, and themselves, justice, by its speedy possession and careful study. We will venture to say one word only in commendation of the literary merits of the work, of which the style and diction proclaim its author to be not only a skilled gynæcologist but an accomplished scholar. H.

*Clinical Lectures on Diseases of the Nervous System.* By WILLIAM A. HAMMOND, M.D., Professor of Diseases of the Mind and Nervous System in the University of New York, etc., etc., etc. Reported, edited, and the histories of the cases prepared, with notes, by T. M. B. CROSS, M.D., Assistant to the Chair of Diseases of the Mind and Nervous System in the University of New York. New York: D. Appleton & Co. 1874.

Dr. Hammond's Treatise on Diseases of the Nervous System, has become so universally popular as to be familiar to most of our readers, who will be glad to accept this effort to condense the essential propositions embodied in that work, into the less bulky proportions of this one just issued, by which the author's views are brought more directly within the reach of a much greater number, who may have been deterred by its greater bulk and cost from the study of the former work. While the volume before us is to some extent a condensed summary of the principles of the larger treatise, it is something more than this, for while many of the subjects of lesser importance are omitted, others of more directly practical value have been elaborated more fully, to adapt them more especially to the necessities of a clinical class.

Of Dr. Hammond's acquirements as a neuropathologist, as a clinical teacher, or as a scholar, it is needless to speak here, but we will take the liberty to felicitate him upon his good fortune and judgment in the selection of his assistant and editor, Dr. T. M. B. Cross, to whom both he and the profession at large are equally indebted for his labors in the collation of the data and the preparation of these lectures. H.

### BOOKS RECEIVED.

*Erysipelas, and Child Bed Fever.* By THOMAS C. MINOR, M.D. Cincinnati: Robert Clarke & Co. 1874.

*Infant Diet.* By A. JACOBI, M.D., Clinical Professor of Diseases of Children, College of Physicians and Surgeons, New York. Revised and enlarged, and adapted to popular use, by M. P. JACOBI, M.D. Geo. P. Putnam's Sons.

*A Practical Treatise on the Diseases of Women.* By T. GAILLARD THOMAS, M.D., Professor of Obstetrics and Diseases of Women and Children in the College of Physicians and Surgeons, New York; etc., etc. Fourth Edition, thoroughly revised, with one hundred and ninety-one illustrations on wood. Philadelphia: Henry C. Lea. 1874.

*Transactions of the Pathological Society of Philadelphia.* Vol. IV. Containing Report of the Proceedings for the years 1871, '72, '73. Edited by JAMES TYSON, M.D., Hospital Lecturer on Pathological Anatomy and Histology in the University of Pennsylvania, etc., Recorder of the Society. Philadelphia: J. B. Lippincott & Co. 1874.

*Essentials of the Principles and Practice of Medicine.* A Hand-Book for Students and Practitioners. By HENRY HARTSHORNE, A.M., M.D., Professor of Hygiene in the University of Pennsylvania, etc., etc. Fourth Edition, thoroughly revised; with one hundred illustrations. Philadelphia: Henry C. Lea. 1874.

*The Nature of Gunshot Wounds of the Abdomen, and their Treatment:* based on a review of the Case of the late James Fisk, Jr., in its Medico-Legal Aspects. By EUGENE PEUGNET, M.D., Surgeon to the Northwestern Dispensary; Member of the New York Pathological Society, etc., etc. New York: William Wood & Co. 1874.

PAMPHLETS RECEIVED.

*Transactions of the Medical Association of the State of Alabama.* Twenty-Seventh Session, 1874.

*A Case of Anchylosis of Right Temporo-Maxillary Articulation,* Successfully Treated by Excision of the Condyle; with Remarks. By JAMES L. LITTLE, M.D., Surgeon to St. Luke's Hospital, etc., etc. Albany, 1874.

*Corpulency, and its New Self-Dietary Cure.* Consisting of letters to the Medical Times and Gazette, explaining his newly discovered Diet-System to reduce the weight and benefit the health. By A. W. MOORE, Member of the Royal College of Surgeons and L.S.A. Sixth Edition. Printed for the Author by Spottiswoode & Co., New-Street Square, London.

*Injuries of the Skull.* By C. C. F. GAY, M.D.

*Transactions of the Medical Society of the State of Pennsylvania,* at its Twenty-Fifth Annual Session, held at Easton, Pa., May, 1874. Vol. X. Part I. 1874.

*The Address in Obstetrics delivered before the Medical Society of the State of Pennsylvania,* May, 1874. By WILLIAM B. ATKINSON, Physician to the Department of Obstetrics and Diseases of Women, Howard Hospital, Philadelphia. 1874.

*The Ophthalmoscopic Appearances of the Optic Nerve in Cases of Cerebral Tumor.* By C. E. Fitzgerald, M.D., C.H.M., Dublin, etc. Dublin: Fannin & Co., 41 Grafton street. London: Longmans & Co. 1874.

*Tinnitus Aurium, Noises in the Ears.* By LAWRENCE TURNBULL, M.D., Physician to the Department of Diseases of the Eye and Ear, Howard Hospital, Philadelphia. Philadelphia: J. B. Lippincott & Co. 1874.

*Deaf Muteism, and the Method of Educating the Deaf and Dumb.* By LAWRENCE TURNBULL, M.D.

JOURNALS RECEIVED.

The American Medical Weekly, Sept. 21, 29, Oct. 3, 10, 17.
" Australasian Medical and Surgical Review—July, 1874.
" Archives of Dermatology—Vol. 1, No. 1, October, 1874.
" Atlanta Medical and Surgical Journal—October.
" American Practitioner—October.

Boston Medical and Surgical Journal—October, 1874.
Boston Journal of Chemistry—October.
The Clinic, Cincinnati—September 19, Oct. 3, 10, 17.
" Canada Lancet—October.
" Druggists' Circular—October, 1874.
" Detroit Review of Medicine and Pharmacy—October, 1874.
" Indiana Journal of Medicine—October.
" Kansas City Medical Journal—September and October.
" London Lancet—September, 1874.
" Medical Times, Philadelphia—Sept. 19, Oct, 3, 10, 17.
" Medical and Surgical Reporter—Philadelphia, Sept. 19, Oct. 3, 10, 17.
" Missouri Clinical Record—October.
" Medical Record, New York—October 1.
" Medical News and Library—October, 1874.
" " Supplement, Oct., 1874.
" Medical Herald, Leavenworth, Kansas—October.
" Medical Examiner, Chicago—October 15.
" New York Medical Journal—October, 1874.
" Nashville Journal of Medicine and Surgery—October.
" Practitioner, London—Sept., 1874.
" Pacific Medical and Surgical Journal—Sept., 1874.
" Pharmacist, Chicago—October, 1874.
" Progres Medicale—Nos. 36, 37, 38, 39, 40.
" Richmond and Louisville Medical and Surgical Journal—Sept., 1874.
" St. Louis Medical and Surgical Journal, Oct., 1874.
" Southern Medical Record—September.
" Transactions of the Wisconsin State Medical Society—1874.
" Texas Medical Journal—August, 1874.
" Virginia Medical Monthly—October, 1874.

---

